# Monitoring inflammation injuries in the progression of atherosclerosis with contrast enhanced ultrasound molecular imaging

**DOI:** 10.1371/journal.pone.0186155

**Published:** 2017-10-05

**Authors:** Ruiying Sun, Jie Tian, Jun Zhang, Liping Wang, Jing Guo, Yani Liu

**Affiliations:** Department of Medical Ultrasound, Tongji Hospital, Tongji Medical College, Huazhong University of Science and Technology, Wuhan, Hubei, China; Monash University, AUSTRALIA

## Abstract

**Purpose:**

The upregulation of vascular cell adhesion molecule-1(VCAM-1) on vascular endothelium plays a great role in the progression of atherosclerosis (AS). In this study, ultrasound molecular imaging was performed to monitor the inflammation injuries in the onset and progression of atherosclerosis with microbubbles targeted to VCAM-1.

**Methods:**

Mice deficient for the apolipoprotein E (ApoE^-/-^mice) with high-cholesterol diet were studied as an age-dependent model of atherosclerosis. At 8, 16, 24, and 32 weeks of age, contrast enhanced ultrasound (CEU) molecular imaging of proximal ascending aorta was performed with microbubbles targeted to VCAM-1. Plaque size, monocytes infiltration and the expression of VCAM-1 in the proximal ascending aorta were assessed by histology and western blot analysis, separately.

**Results:**

In ApoE^-/-^ mice, molecular imaging for VCAM-1 detected selective signal enhancement (P<0.01 versus non-targeted microbubbles) at all ages of ApoE^-/-^ mice. Moreover, signals from targeted microbubbles increased from 8wks to 32wks age (P<0.05 for trend) in ApoE^-/-^ mice, indicating the upregulation of VCAM-1 with the progression of atherosclerosis. Consistent with CEU imaging results, both western blot analysis and immunohistochemistry revealed the expression of VCAM-1 and monocytes infiltration were age-dependent in ApoE^-/-^ mice.

**Conclusions:**

CEU molecular imaging can be used to noninvasively detect the VCAM-1 expression on the endothelium in the progression of atherosclerosis. By investigating specific molecular biomarkers, it could help to monitor the inflammation and the progression of AS, which may in some extent contribute to the prediction of vulnerable plaque.

## Introduction

It is widely accepted that inflammation plays a key regulatory role in the pathogenesis of atherosclerosis (AS) and its complications [1–3]. Different types of inflammatory responses, including the upregulation of adhesion molecules, recruitment and differentiation of monocyte/macrophage, and the release of inflammatory cytokines and chemokines, are all involved in the initiation and progress of AS [2–3]. More importantly, accumulating evidence revealed the close link between inflammation and plaque rupture and thrombosis, which accounts for the majority of cardiovascular and cerebrovascular events [4–6]. Based on the basic biology of inflammation in AS, it is quite urgent and important to precisely evaluate inflammatory severity in vivo, which contributes to cardiovascular risk prediction, as well as AS monitoring and therapy evaluation.

In recent years, a growing experimental literature demonstrated that contrast enhanced ultrasound (CEU) can be used to noninvasively image inflammatory activity with microbubbles (Mb) specific to the inflammatory markers on the endothelium [7–9]. Among the specific molecular targets for inflammation, vascular cell adhesion molecule-1 (VCAM-1), expressed on activated endothelial cells, is regarded as an ideal target for CEU imaging because there is no or less constitutive expression in normal conditions [10–12]. In fact, VCAM-1 not only plays an important role in the onset of AS by recruiting monocytes and lymphocytes to the arterial intima. Also it participates in the ongoing inflammatory cascade and the complete course of vulnerable plaque development [13–14]. In the present study, CEU molecular imaging was performed to quantify inflammation severity in AS with VCAM-1 targeted microbubbles. Specifically, the dynamics process of inflammation and the progression of AS from the initial to the advanced plaque were monitored in vivo.

## Materials and methods

### Animal models

All the experimental operations of animals were performed in accordance with the protocols approved by the Institutional Ethical Committee of Tongji Hospital,Tongji Medical College, Huazhong University of Science and Technology. All animals were treated humanely according to the guidelines of the Institutional Ethical Committee of Tongji Hospital,Tongji Medical College, Huazhong University of Science and Technology.The study was approved by the above-mentioned Ethical Committee and the approval number is TJ-A20150201.Fifty-four control wild-type C57BL/6J mice and sixty-two apolipoprotein E deficient mice (ApoE^-/-^) on C57BL/6J background underwent imaging studies at 8, 16, 24 and 32 weeks of age (n = 13 to 20 for each strain at each age) (Beijing HFK Bioscience Co.,Ltd). From 8 weeks of age on, ApoE^-/-^ mice were fed on a hypercholesterolemic diet (containing 21% fat by weight, 0.15% cholesterol and 19.5% casein) and the serum lipid profile was analyzed (n = 5 for each strain at each age). After imaging study, all the mice were sacrafied for aortic histology and western blot analysis.

### Microbubble preparation

Biotinylated, lipid-shelled decafluorobutane microbubbles were prepared by sonication of a gas-saturated aqueous suspension of distearoylphosphatidylcholine, polyoxyethylene-40-stearate, and distearoylphosphatidylethanolamine-PEG(2000) biotin [15]. Rat antimouse monoclonal IgG1 against VCAM-1 (MK 2.7) (BD bioscience) and rat isotype control antibody (BD bioscience) were conjugated to the surface of microbubbles to produce VCAM-1–targeted (MBv) and control (MBc) microbubbles as previously described [15]. Microbubble concentration and size distribution were measured by electrozone sensing (Multisizer III, Beckman-Coulter).

### CEU molecular imaging

Mice were anesthetized with inhaled isoflurane (1.0–1.5%). A jugular vein was cannulated for microbubble administration. Contrast-enhanced ultrasound imaging (Sequoia, Siemens Medical System) of ascending aorta and the origin of brachiocephalic trunk from right sternal window was performed with a high-frequency linear-array probe (15L8). Power modulation and pulse inversion imaging at a frequency of 7MHz and a dynamic range of 50dB was performed. The gain setting was adjusted to levels just below visible speckle and held constant. VCAM-1 targeted or control microbubble (1×10^6^ per injection) was injected intravenously in random order. For the detection of the specific contrast agent, ultrasound imaging was frozen from injection until 8 minutes later when imaging was resumed at the mechanical index of 0.97. The first 20 frames images, which were used to derive the total amount of microbubbles within the ascending aorta and the origin of brachiocephalic trunk, were acquired at a pulsing interval (PI) of 1s. Then mechanical index was briefly increased to 1.9 to completely destroy microbubble in the imaging field. Subsequent 10 frames were acquired at a PI of 5 s to measure the signal from freely circulating microbubbles.

The signals from microbubbles were quantitatively estimated by video intensity analysis software (iMCE). As previously described [15], frames representing freely circulating microbubbles were digitally subtracted from the first 20 frame images to derive the signal from attached microbubbles. The region of interest (ROI) was placed on the ascending aorta and the origin of brachiocephalic artery, which was guided by fundamental 2-dimensional imaging at 14 MHz.

### High-frequency ultrasound imaging

High-frequency (30 MHz) ultrasound imaging (Vevo 2100, VisualSonics Inc, Toronto, Canada) was used to assess left ventricle (LV) function and plaque size on parasternal views. Left ventricular fractional shortening and LV ejection fraction were assessed in the midventricular short-axis plane. Stroke volume was calculated by the product of left ventricle outflow tract area and left ventricle outflow tract time-velocity integral on pulsed-wave Doppler. Aortic centerline velocity was measured by pulsed-wave Doppler in the distal aortic arch.

Plaque size was assessed by measuring vessel wall thickness at lesion-prone sites of the lesser curvature of the aortic arch and the origin of the brachiocephalic artery (near wall) at end-diastole.

### Histology

Perfusion fixation was performed and short axis sections of the ascending aorta were paraffin embedded providing 3 separate regions for each subject. Masson trichrome staining was performed to assess the plaque area calculated by the vessel tissue area within the internal elastic lamina, which reflect the severity of atherosclerotic lesion. Immunohistochemistry was performed using goat polyclonal primary antibodies against mouse Mac-2 (M3/38, eBioscience) for identifying monocytes and macrophages infiltration. Species-appropriate ALEXAFluor-488 secondary antibody (Invitrogen Grand Island, NY) was used. Spatial extent of Mac-2 expression was quantified by using nonconfocal microscopy, which provided the optimal binary data for positive/negative staining using Image-J software (version 1.48, National Institutes of Health) and a threshold of >2 SD above of the tunica media intensity, excluding the elastic lamina. Data were expressed as Mac-2–positive area within the internal elastic lamina. Western blot analysis was performed to determine the expression of VCAM-1 in the ascending aorta (n = 5 mice in each group). Goat polyclonal antibody against VCAM-1 (Santa Cruz) and anti-GAPDH mouse monoclonal antibody (Boster Biological Technology, Wuhan, China.) as internal control were used as a primary antibody with a horse radish peroxidase (HRP) labeled secondary antibody (Boster Biological Technology, Wuhan, China.). Optical density value and the relative grey value represented the expression of VCAM-1 protein.

### Statistical analysis

Data was analyzed on SPSS (version 17.0), all parametric data was expressed as mean±SD. Homogeneity test for variance was used when appropriate. Comparisons between C57BL/6J and ApoE^-/-^ mice of each age group were made with unpaired Student *t* test (2-tailed). Comparisons between different age cohorts within the same animal group were made with 1-way ANOVA and unpaired Student *t* test, differences were considered significant at P<0.05. Bonferroni correction was applied for multiple comparisons. A Spearman rank correlation test was used to assess the relationship between age and histology data.

## Results

### Lipid profile and left ventricular function

ApoE^-/-^ mice had higher serum cholesterol (8W: 5.0 fold; 16W: 8.7 fold, 24W: 18.5 fold, 32w: 25.0 fold) and much higher LDL (8W: 4.5 fold; 16W: 21.0 fold, 24W: 56.0 fold, 32w: 61.9 fold) than C57BL/6J mice at each age group (Table 1). In ApoE^-/-^ mice, there were progressive increases of the serum cholesterol, triglyceride and LDL levels from 8 weeks to 32 weeks. For C57BL/6J mice, there was no significant difference among these parameters from 8 weeks to 32 weeks (Table 1).

**Table 1:** Serum lipid profile data (Mean±SD).

	ApoE deficient mice				C57BL/6J mice			
	ApoE^-/-^ 8WK	ApoE^-/-^ 16WK	ApoE^-/-^ 24WK	ApoE^-/-^32WK	C57BL/6J 8WK	C57BL/6J 16WK	C57BL/6J 24WK	C57BL/6J 32WK
Total Cholesterol, CHOL (mmol/L)	6.9±1.34	19.94±1.50*	35.54±2.93#	48.18±1.1^△^	1.36±0.14*	2.29±0.40#	1.92±0.16^△^	1.88±0.13†
Triglycerides, TG (mmol/L)	0.64±0.18	1.29±0.14*	1.89±1.09#	2.66±1.18^△^	0.63±0.18	0.83±0.10#	1.62±0.18^△^	1.1±0.22†
Low-density lipoprotein, LDL (mmol/L)	0.54±0.2	6.73±0.27*	7.29±3.88#	11.14±0.76^△^	0.12±0.01*	0.32±0.13#	0.13±0.04^△^	0.18±0.09†
High-density lipoprotein, HDL (mmol/L)	0.43±0.15	0.21±0.07*	0.21±0.12	0.18±0.10^△^	0.88±0.09*	1.21±0.17#	1.18±0.14^△^	1.12±0.03†

ApoE indicates apolipoprotein E

* *P*<0.05 versus ApoE^-/-^ 8WK

^#^*P*<0.05 versus ApoE^-/-^ 16WK

^△^*P*<0.05 versus ApoE^-/-^ 24WK

^†^*P*<0.05 versus ApoE^-/-^32WK

On echocardiography, left ventricular fractional shortening, left ventricular ejection fraction, and stroke volume were not significantly different for C57BL/6J and ApoE^-/-^ mice at each age (Table 2). Aortic peak systolic flow velocities were not different among all the groups suggesting similar hemodynamic conditions and shear rates, which could potentially influence targeted microbubble attachment.

**Table 2:** Hemodynamic and echocardiography data (mean±SD).

	ApoE deficient mice				C57BL/6J mice			
	ApoE^-/-^ 8WK	ApoE^-/-^ 16WK	ApoE^-/-^ 24WK	ApoE^-/-^ 32WK	C57BL/6J 8WK	C57BL/6J 16WK	C57BL/6J 24WK	C57BL/6J 32WK
Fractional shortening, %	39.23±3.12	40.87±1.83	37.69±3.56	41.73±4.88	37.89±4.51	39.55±3.78	38.78±2.33	42.13±2.21
LVEF, %	70.51±3.38	70.82±1.90	68.15±4.54	72.04±5.00	69.24±4.66	71.57±3.24	70.56±3.45	73.43±2.05
Stroke volume, μL	33.78±4.23	33.00±5.29	34.25±4.81	36.47±5.31	34.61±3.86	37.01±2.63	37.36±1.99	37.94±3.38
Aortic peak systolic velocity, m/s	0.69±0.21	0.69±0.23	0.72±0.11	0.73±0.07	0.65±0.26	0.70±0.18	0.71±0.08	0.72±0.15

ApoE indicates apolipoprotein E

### Vessel morphometry and histology

**Fig 1:**
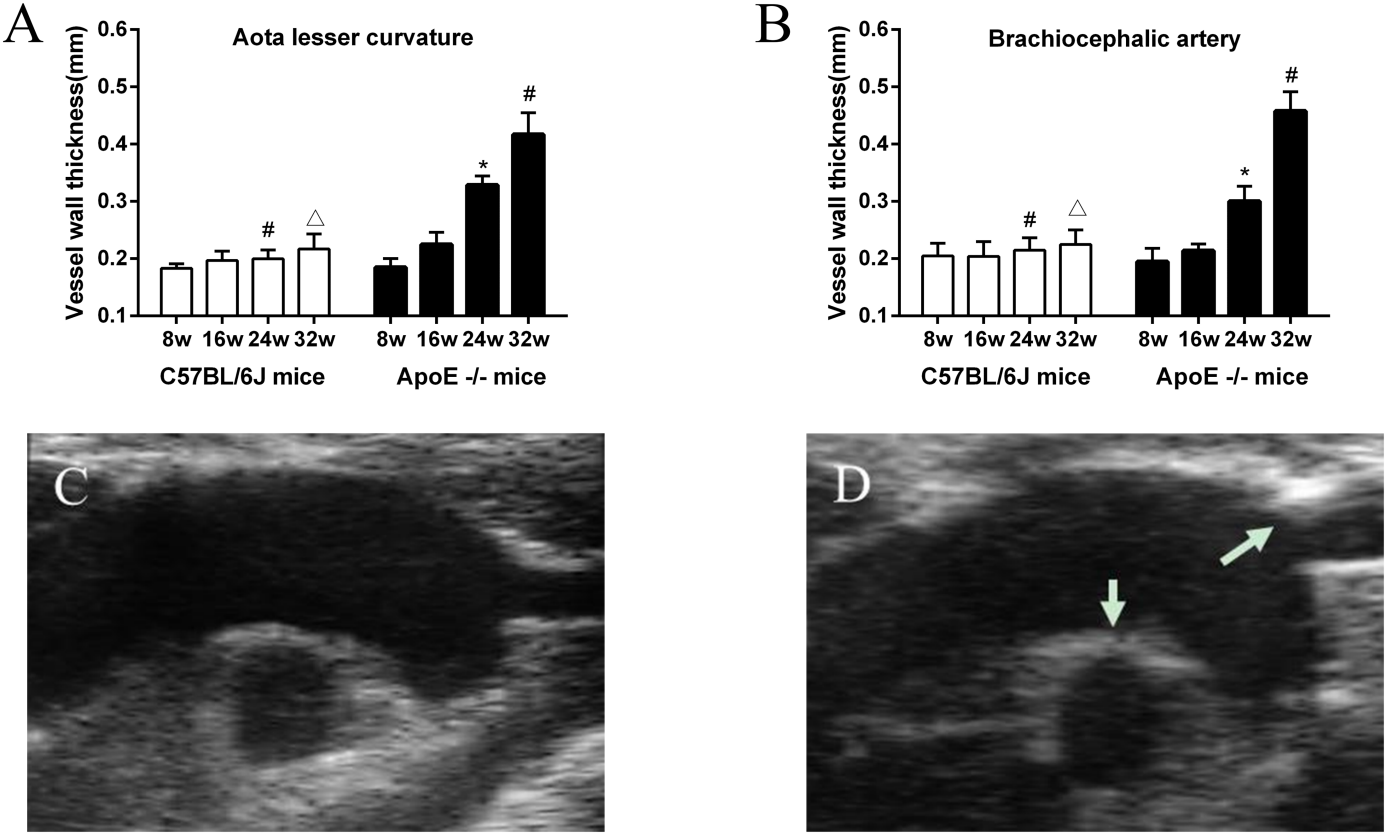
Vessel wall thickness (mean±SEM) measured by high-frequency ultrasound imaging of the aortic arch measured at (A) the lesser curvature of the aortic arch, and (B) at the origin of the brachiocephalic artery. Images illustrate examples obtained from C57BL/6J(C) and ApoE-/- mouse (D) at 32 weeks. In the ApoE-/- mouse, there was a focal plaque detected in the proximal brachiocephalic artery (white arrow) and severe thickening of the lesser curvature (white arrows). *P<0.05 versus ApoE-/- 16WK. #P<0.05 versus ApoE-/- 24WK. △P<0.05 versus ApoE-/- 32WK.

On high-frequency ultrasound, vessel thickness at the lesion prone sites of the lesser curvature of the aorta and the proximal brachiocephalic artery did not change with age in C57BL/6J mice. In ApoE^-/-^ mice, vessel wall thickness increased from 8 weeks to 32 weeks of age, consistent with the age-dependent worsening of atherosclerosis in ApoE^-/-^ model. Focal plaque could be detected in the brachiocephalic artery in most of ApoE^-/-^ mice at 32 weeks of age. The significant difference of the vessel wall thickness between ApoE^-/-^ and C57BL/6J mice was only detected at 24 and 32 weeks of age, indicating that the small lesions seen on histology could not be reliably detected by high-frequency ultrasound (Fig 1).

**Fig 2:**
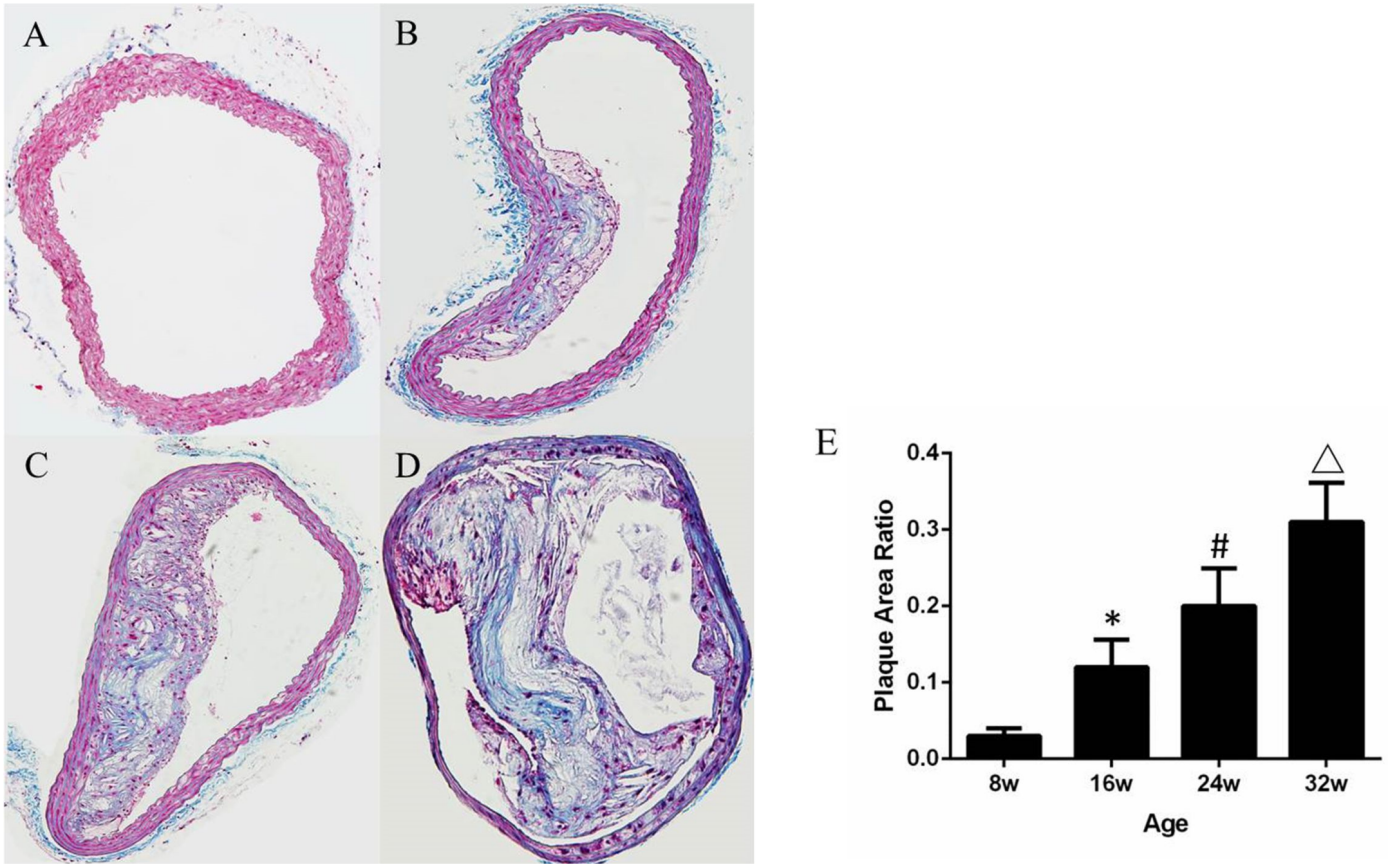
Masson trichrome stains of the ascending aorta in ApoE-/- mice. (A) Image from ApoE-/- mice at 8 weeks showed mild intimal thickening and sparse monocytes adhesion to the endothelium; (B) Small plaques was detected at 16 weeks; (C) Typical plaques with lipid core formation at 24 weeks; (D) Big plaques with lipid-rich core, necrosis region and inflammatory cells infiltration at 32 weeks. (E) Mean (±SEM) plaque area (ratio to the vessel area) increased with age for ApoE-/- mice (Spearman rank correlation coefficient 0.92; P<0.001). * P<0.05 versus ApoE-/- 8WK. #P<0.05 versus ApoE-/- 16WK. △P<0.05 versus ApoE-/- 24WK. †P<0.05 versus ApoE-/- 32WK.

On histology, there was no evidence for plaque development in C57BL/6J mice at any age. In ApoE^-/-^ mice, there were regions of mild intimal thickening and sparse monocytes adhesion to the endothelium detected in some of the animals at 8 weeks of age. Small but discrete fibrous plaques were seen at 16 weeks of age. All sections from 24 weeks old ApoE^-/-^ mice demonstrated typical plaques with lipid core in aorta (Fig 2). At 32 weeks of age, big plaques with lipid-rich core, necrosis region and inflammatory cells infiltration were seen in all the sections, and these lesions tended to protrude into the aortic lumen (Fig 2). The ratio of plaque to vessel wall area in ApoE^-/-^ mice increased from 8 to 32 weeks (Spearman rank correlation coefficient 0.92; P<0.001), indicating the progression of atherosclerosis in ApoE^-/-^ mice (Fig 2).

### Targeted imaging of VCAM-1 expression in the progression of AS

**Fig 3:**
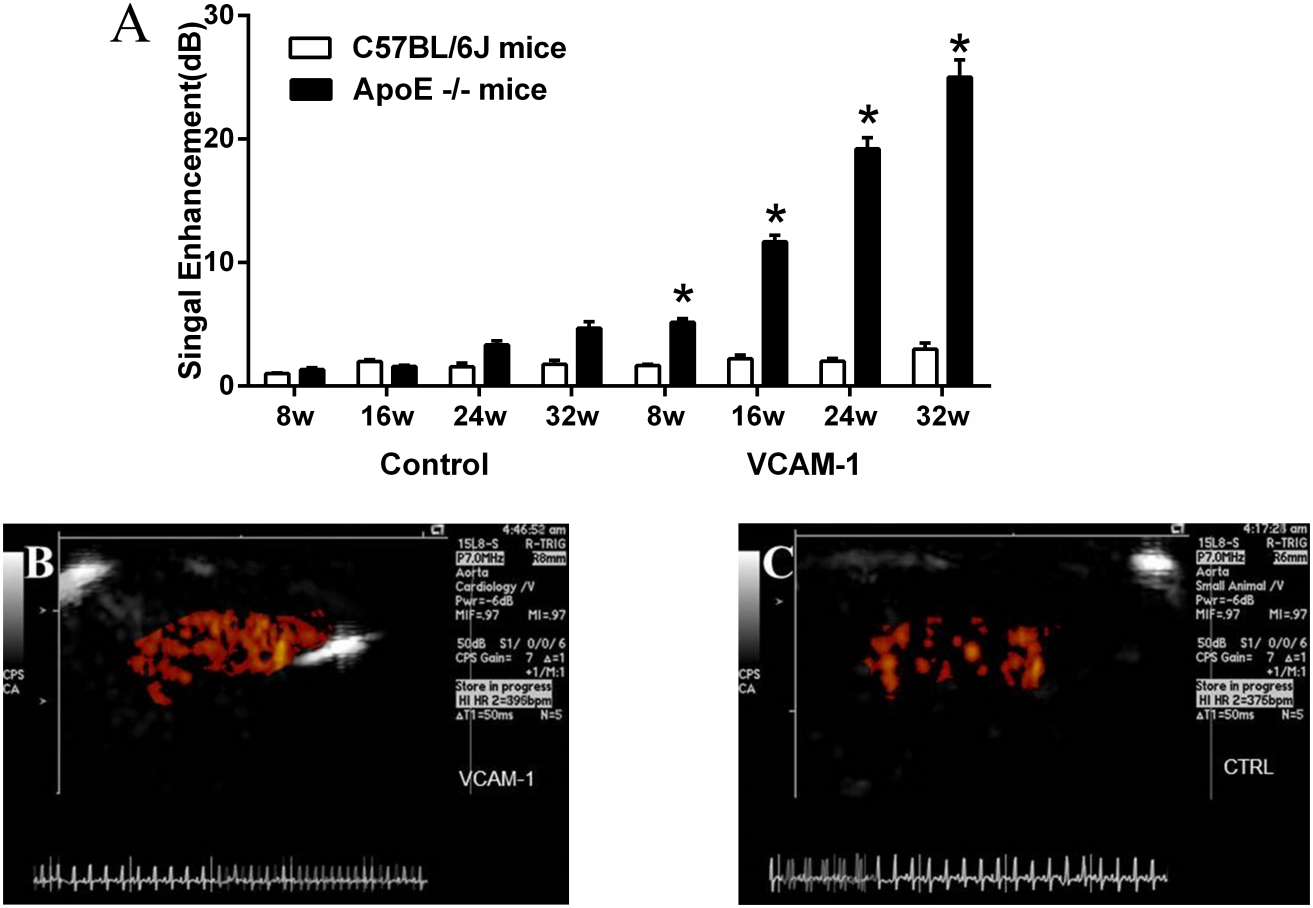
A. Molecular imaging signal intensity (mean±SEM) for VCAM-1 and for control nontargeted microbubbles. Examples of molecular imaging of the ascending aorta and arch in a 32 weeks of age ApoE-/- mouse with (B) VCAM-1–targeted, (C) control microbubbles. * P<0.05 versus control microbubble and compared to corresponding data in wild-type mice.

In ApoE^-/-^ mice, CEU molecular imaging of the ascending aorta and arch detected selective signal enhancement for VCAM-1 targeted microbubbles compared to control microbubbles at 8, 16, 24 and 32 weeks of age. Moreover, signal from VCAM-1 targeted microbubbles increased from 8 to 32 weeks of age (P<0.05 for trend). VCAM-1 signals in ApoE^-/-^mice were greater than wild-type controls at all time points. In C57BL/6J wild-type mice, there was no statistically difference in CEU molecular imaging signal between control microbubbles and VCAM-1 targeted microbubbles (Fig 3).

### Immunohistochemistry and Western blot analysis

**Fig 4:**
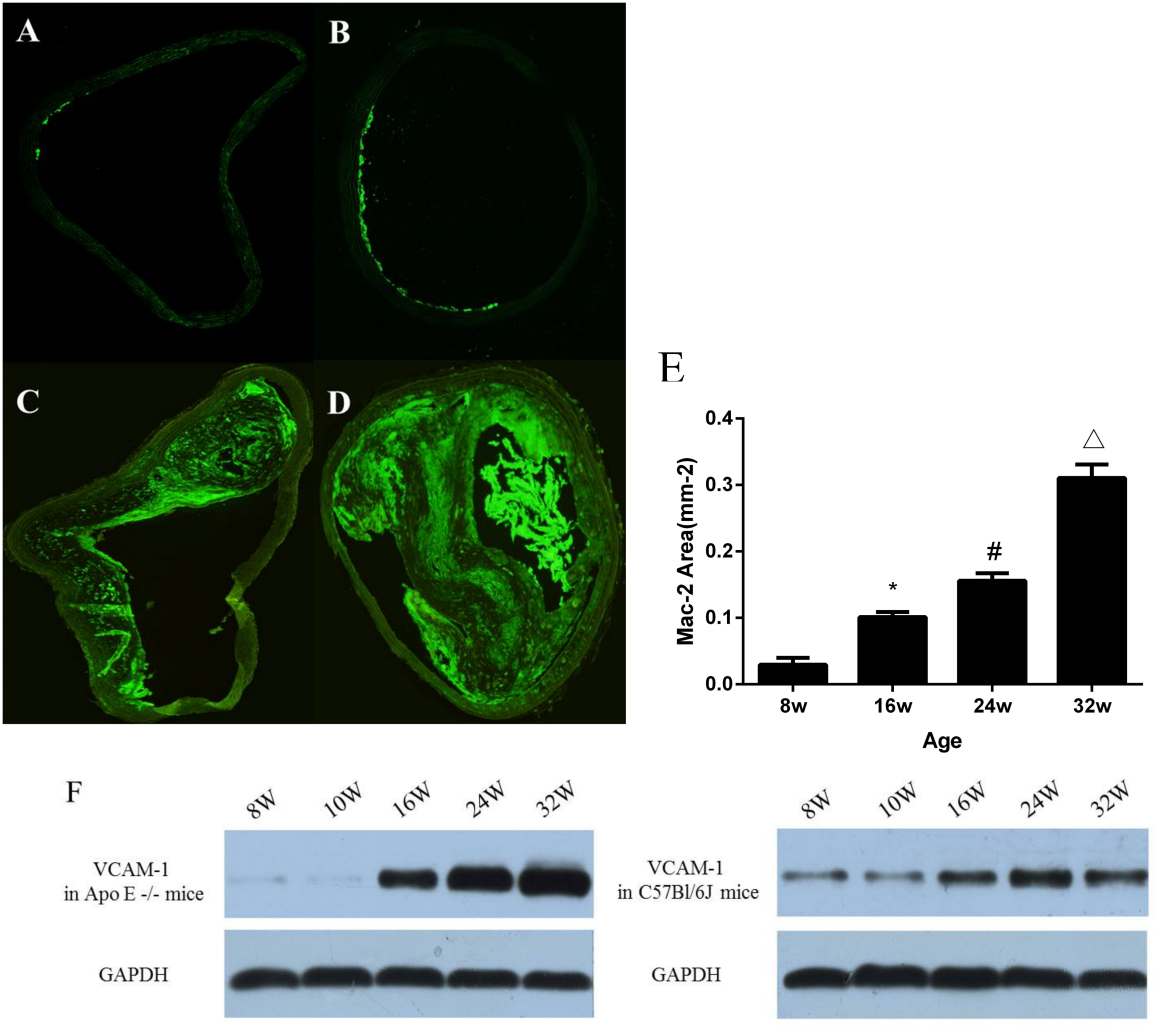
Mac-2 staining illustrating differences monocyte/macrophage accumulation in the different age groups of Apo E -/- mice. (A) 8 weeks of age; (B) 16 weeks of age; (C) 24 weeks of age; and (D) 32 weeks of age. (E) Mean (±SEM) area staining positive for Mac-2 increased with age in ApoE-/- mice (Spearman rank correlation coefficient 0.89; P<0.001). (F) Western blot analysis of VCAM-1 expression of the ascending aorta in ApoE-/- and C57BL/6J mice. In ApoE-/- mice, VCAM-1 expression in the ascending aorta increased with the age.

On immunohistochemistry for Mac-2, there was no positive staining in wild-type mice at all ages. In ApoE^-/-^ mice at 8 and 16 weeks of age (Fig 4A and 4B), minimal and local Mac-2-positive cells were present on the intimal surface of the aorta. The monocyte infiltration increased with the age. There was abundant Mac-2–positive cells (monocytes and macrophages) infiltration in atherosclerotic lesions of ApoE^-/-^ mice at 24 and 32 weeks of age (Fig 4C and 4D). The spatial extent of Mac-2 expression increased with age in ApoE^-/-^ mice (Fig 4E) (Spearman rank correlation coefficient 0.89; P<0.001).

Consistent with molecular imaging results (Fig 4F), Western Blot analysis revealed the age-dependent increase of VCAM-1 expression in the ascending aorta in ApoE^-/-^ mice. And there was only minimal VCAM-1 expression in C57BL/6J mice at all ages.

## Discussion

In the present study, CEU molecular imaging was performed in a reproducible age-dependent murine model of aortic atherosclerosis. Our study showed that pathogenic VCAM-1 expression measured by CEU molecular imaging increased progressively from 8 to 32 weeks of age, which was consistent with atherosclerotic lesions progression. We also found the same increase trend for both specific signal enhancement from VCAM-1 targeted microbubbles in CEUS imaging and the monocytes/macrophages accumulation assessed by Mac-2 staining. These findings indicated that noninvasive CEU molecular imaging could detect the initial inflammatory response before the appearance of the advanced plaques, also can be used to monitor the inflammation and the progression of AS.

It has been widely accepted that atherosclerosis is a chronic inflammatory disease and inflammatory response plays a critical role in the initiation, progression and rupture of the atherosclerotic plaques [1–4]. In recent years, many noninvasive imaging modalities, including ultrasound, magnetic resonance imaging (MRI) and positron emission tomography (PET) were developed to prepare new targeted contrast agents to visualize inflammation process in atherosclerosis [10–11,16–17]. Compared to the other imaging modalities, ultrasound is often set to be the first choice in vascular imaging because it is noninvasive and convenient. Different to the traditional ultrasound imaging which is focusing on anatomy, CEU molecular imaging is more applied to detect specific molecular phenotype in vivo with microbubbles bearing specific ligand [7–9]. Lindner used P-selectin targeted microbubbles to detect the early endothelial injuries in transplant rejection [7]. Davidson used selectins targeted microbubbles for imaging recent myocardial ischemia [18]. Although some previous studies demonstrated that CEU molecular imaging can be used to detect the specific inflammatory markers with targeted microbubbles [7–9, 12], few studies have continuously monitor the progression of the inflammatory markers in the complete course of atherosclerosis development. To this purpose, noninvasive CEU molecular imaging was performed to continuously evaluate inflammatory severity and to monitor the change of inflammation response during the complete course of plaque development.

This study was performed in an age-dependent murine model of atherosclerosis that could develop complex lesions of atherosclerosis similar to those in humans. Within the last two decades, the histological features and cellular composition of atherosclerotic lesions in mice deficient for the ApoE mice models have been fully studied [19–20]. In the present study, histology demonstrated lesions progressing from sparse mild intimal thickening at 8 weeks to widespread atherosclerotic plaques with luminal encroachment at 32 weeks, indicating a reproducible age-dependent worsening of lesions.

In the progression of atherosclerosis, a large amount of inflammatory cellular and molecular players have been identified that promote plaque initiation, maturation and rupture [2–4]. In our study, VCAM-1 expressed on the endothelium was chosen as the inflammatory target for molecular imaging because of its function to recruit monocytes and lymphocytes to the arterial intima in the early stage of atherosclerosis [14]. As the disease advances, monocytes and macrophages could secrete abundant growth factors, which promote the more expression of adhesion molecules and recruit more monocytes adhering to endothelium, as a result of amplifying the inflammatory response [10–11]. Nakashima analyzed the expression of VCAM-1 and intercellular adhesion molecule-1 (ICAM-1) en face on the aortic endothelium of ApoE^-/-^ mice. In his study, expression of VCAM-1 preceded lesion formation, and increased expression above control levels appeared to be correlated with the extent of exposure to plasma cholesterol [13]. Kaufmann not only confirmed the selective attachment of VCAM-1 targeted microbubbles to aortic plaque in ex vivo studies [12]. Also his study indicated that CEU molecular imaging was capable of quantifying aortic VCAM-1 expression with microbubbles targeted to VCAM-1 in vivo [12]. In the present study, we further found that CEU molecular imaging with microbubbles targeted to VCAM-1 could be used to reveal the early molecular event which precedes plaque formation. More importantly, specific signal enhancement from targeted microbubbles increased with age in ApoE^-/-^ mice, which correlated with the extent of plaque formation, monocytes/macrophages infiltration and VCAM-1 expression in aorta. Our findings demonstrated the CEU molecular imaging has the potential to monitor the inflammatory activity throughout the atherosclerotic lesion development.

Nowadays, identification of a plaque more likely to rupture remains a greatest challenge in cardiovascular medicine [21].A growing number of scholars and researchers begin to work on anti-inflammatory modalities such as statins, anti-oxidative therapy, and inhibiting lipoprotein associated phospholipase A2, aimed to attenuate or halt progression of atherosclerosis [16, 22–23].Therefore, noninvasively monitoring the changes in arterial inflammation appears quite importantly during the long-term therapeutics for modulation of atherosclerosis [24].In our study, the most remarkable signal enhancement from VCAM-1 targeted microbubbles was detected in ApoE^-/-^ mice at 32 week of age, in which a large amount of big plaques with lipid-rich core, necrosis region and inflammatory cells infiltration were detected with Masson’s trichrome staining. Besides, the overexpression of VCAM-1 in ApoE^-/-^ mice at each age was concomitent to the presence of macrophages on Mac-2 staining, thereby indicative of the occurrence of an inflammatory process similar to that observed in vulnerable lesions. The directly proportional relationship between the VCAM-1 expression and the signal enhancement shows an evidence that the CEU molecular imaging is quite promising in monitoring anti-inflammatory treatments by continuously quantifying specific inflammatory biomarkers in vivo.

There are also several limitations of this study though. First, although signal enhancement from control microbubbles was very low in Apo E^-/-^ mice compared with microbubbles targeted to VCAM-1, it still increased in 24 week and 32 week groups. This likely represents low-level interaction of lipid microbubbles with leukocytes on the vascular surface. Second, although the VCAM-1 expression in the ascending aorta which determined by Western Blot analysis could not reflect the exact amount of VCAM-1 on the endothelium accessible to the targeted microbubble, it revealed the same age-dependent increase of inflammation injuries as detected by molecular imaging with microbubbles targeted to VCAM-1.

## Conclusions

In conclusion, our study demonstrated that CEU molecular imaging can be used to noninvasively detect the VCAM-1 expression on the endothelium in the procession of atherosclerosis. By detecting the high-risk plaque molecular biomarkers, CEU molecular imaging has the potential to quantify and monitor the inflammation activity in vivo and over time, which may contribute to the prediction of vulnerable plaque and to assessing the effect of anti-atherosclerosis therapies that aim to stabilize vulnerable plaques and silence vascular inflammation.

## Supporting information

S1 Table Supporting data-thickness. (XLSX)

S2 Table Supporting data-plaqueratio. (XLSX)

S3 Table Supporting data-enhancement. (XLSX)

S4 Table Supporting data-mac2. (XLSX)

S5 Table Supporting data-wb. (XLSX)

## Abbreviations

ApoE^-/-^: mice deficient for the apolipoprotein E
AS: atherosclerosis
CEU: contrast enhanced ultrasound
HRP: horse radish peroxidase
ICAM-1: intercellular adhesion molecule-1
LV: left ventricle
PI: pulsing interval
ROI: the region of interest
VCAM-1: vascular cell adhesion molecule-1
VH-IVUS: virtual histology intravascular ultrasound imaging

## References

[1] Ross R.Atherosclerosis-an inflammatory disease.N Engl J.Med.1999;340 (2): 115–126. doi: 10.1056/NEJM199901143400207 9887164 10.1056/NEJM199901143400207

[2] Libby P, Okamoto Y, Rocha VZ, Folco E. Inflammation in atherosclerosis: transition from theory to practice. Circ J. 2010;74(2):213–220. doi: 10.1253/circj.CJ-09-0706 20065609 10.1253/circj.cj-09-0706

[3] Soeki T, Sata M. Inflammatory Biomarkers and Atherosclerosis. Int Heart J. 2016;57(2):134–139. doi: 10.1536/ihj.15-346 26973275 10.1536/ihj.15-346

[4] Bentzon JF, Otsuka F, Virmani R, Falk E. Mechanisms of plaque formation and rupture. Circ Res. 2014;114(12):1852–1866. doi: 10.1161/CIRCRESAHA.114.302721 24902970 10.1161/CIRCRESAHA.114.302721

[5] Zhang MD, Zhao XC, Zhang YH Yan YF, Wang ZM, Lv SZ, et al Plaque Thrombosis is reduced by attenuating plaque inflammation with pioglitazone and is evaluated by fluorodeoxyglucose positron emission tomography. Cardiovasc Ther.2015;33(3):118–126. doi: 10.1111/1755-5922.12119 25825053 10.1111/1755-5922.12119

[6] Brunetti ND, Correale M, Pellegrino PL, Munno I, Cuculo A, De Gennaro L, et al Early inflammatory cytokine response: a direct comparison between spontaneous coronary plaque destabilization vs angioplasty induced. Atherosclerosis. 2014;236(2):456–460. doi: 10.1016/j.atherosclerosis.2014.07.037 25173071 10.1016/j.atherosclerosis.2014.07.037

[7] Lindner JR, Song J, Christiansen J, Klibanov AL, Xu F, Ley K. Ultrasound assessment of inflammation and renal tissue injury with microbubbles targeted to P-selectin. Circulation. 2001;104(17):2107–2112. doi: 10.1161/hc4201.097061 11673354 10.1161/hc4201.097061

[8] Steinl DC, Lifen Xu, Elham Khanicheh, Ellertsdottir E, Ochoa-Espinosa A, Mitterhuber M, et al Noninvasive Contrast-Enhanced Ultrasound Molecular Imaging Detects Myocardial Inflammatory Response in Autoimmune Myocarditis. Circ Cardiovasc Imaging.2016; 8;9(8). doi: 10.1161/CIRCIMAGING.116.004720 27502060 10.1161/CIRCIMAGING.116.004720

[9] Deshpande N, Lutz AM, Ren Y, Foygel K, Tian L, Schneider M et al Quantification and Monitoring of Inflammation in Murine Inflammatory Bowel Disease with Targeted Contrast-enhanced US. Radiology. 2012;262(1):172–180. doi: 10.1148/radiol.11110323 22056689 10.1148/radiol.11110323 PMC3244669

[10] Bruckman MA, Jiang K, Simpson EJ, Randolph LN, Luyt LG, Yu X, et al Dual-Modal Magnetic Resonance and Fluorescence Imaging of Atherosclerotic Plaques in Vivo Using VCAM-1 Targeted Tobacco Mosaic Virus. Nano Letters. 2014;14(3):1551–1558. doi: 10.1021/nl404816m 24499194 10.1021/nl404816m PMC4169141

[11] Silvola JMU, Virtanen H, Siitonen R, Hellberg S, Liljenbäck H, Metsälä O, et al Leukocyte trafficking-associated vascular adhesion protein 1 is expressed and functionally active in atherosclerotic plaques. Scientific Reports. 2016;6:35089 doi: 10.1038/srep35089 27731409 10.1038/srep35089 PMC5059718

[12] Kaufmann BA, Sanders JM, Davis C, Xie A, Aldred P, Sarembock IJ, et al Molecular imaging of inflammation in atherosclerosis with targeted ultrasound detection of vascular cell adhesion molecule-1.Circulation. 2007,116(3):276–284. doi: 10.1161/CIRCULATIONAHA.106.684738 17592078 10.1161/CIRCULATIONAHA.106.684738

[13] Nakashima Y, Raines EW, Plump AS, Breslow JL, Ross R. Upregulation of VCAM-1 and ICAM-1 at atherosclerosis-prone sites on the endothelium in the ApoE-deficient mouse. Arterioscler Thromb Vasc Biol. 1998;18(5):842–851 doi: 10.1161/01.ATV.18.5.842 9598845 10.1161/01.atv.18.5.842

[14] Huo Y, Hafezi-Moghadam A, Ley K. Role of vascular cell adhesion molecule-1 and fibronectin connecting segment-1 in monocyte rolling and adhesion on early atherosclerotic lesions. Circ Res. 2000;87(2):153–159 doi: 10.1161/01.RES.87.2.153 10904000 10.1161/01.res.87.2.153

[15] Lindner JR, Song J, Christiansen J, Klibanov AL, Xu F, Ley K. Ultrasound assessment of inflammation and renal tissue injury with microbubbles targeted to P-selectin. Circulation. 2001,104(17):2107–2112. 11673354 10.1161/hc4201.097061

[16] Liu YN, Davidson BP, Yue Q, Belcik T, Xie A, Inaba Y, et al Molecular Imaging of Inflammation and Platelet Adhesion in Advanced Atherosclerosis: Effects of Antioxidant Therapy with NADPH Oxidase Inhibition. Circulation Cardiovascular imaging. 2013;6(1):74–82. doi: 10.1161/CIRCIMAGING.112.975193 23239832 10.1161/CIRCIMAGING.112.975193 PMC3575135

[17] Wu Z. Curaj Adelina, Fokong Stanley, Liehn EA, Weber C, Lammers T, et al Rhodamine-loaded intercellular adhesion molecule-1-targeted microbubbles for dual-modality imaging under controlled shear stresses. Circ Cardiovasc Imaging.2013;6(6):974–981. doi: 10.1161/CIRCIMAGING.113.000805 24036383 10.1161/CIRCIMAGING.113.000805

[18] Davidson BP, Chadderdon SM, Belcik JT, Gupta S, Lindner JR. Ischemic memory imaging in nonhuman primates with echocardiographic molecular imaging of selectin expression. J Am Soc Echocardiogr. 2014;27(7):786–793. doi: 10.1016/j.echo.2014.03.013 24774222 10.1016/j.echo.2014.03.013 PMC4065817

[19] Nakashima Y, Plump AS, Raines EW, Breslow JL, Ross R.ApoE-deficient mice develop lesions of all phases of atherosclerosis throughout the arterial tree. Arterioscler Thromb. 1994;14(1):133–140 doi: 10.1161/01.ATV.14.1.133 8274468 10.1161/01.atv.14.1.133

[20] Emini Veseli B. Perrotta P, De Meyer GRA, Roth L, Van der Donckt C, Martinet W, et al Animal models of atherosclerosis. Eur J Pharmacol. 2017; ISSN 0014-2999. doi: 10.1016/j.ejphar.2017.05.010 28483459 10.1016/j.ejphar.2017.05.010

[21] Narula J, Nakano M, Virmani R, Kolodgie FD, Petersen R, Newcomb R, et al Histopathologic characteristics of atherosclerotic coronary disease and implications of the findings for the invasive and noninvasive detection of vulnerable plaques. Journal of the American College of Cardiology. 2013;61(10):1041–1051. doi: 10.1016/j.jacc.2012.10.054 23473409 10.1016/j.jacc.2012.10.054 PMC3931303

[22] Boekholdt SM, de Winter RJ, Kastelein JJ. Inhibition of lipoprotein-associated phospholipase activity by darapladib: shifting gears in cardiovascular drug development: are antiinflammatory drugs the next frontier? Circulation 2008;118(11):1120–1122. doi: 10.1161/CIRCULATIONAHA.108.795195 18779454 10.1161/CIRCULATIONAHA.108.795195

[23] Singh P, Emami H, Subramanian S, Maurovich-Horvat P, Marincheva-Savcheva G, Medina HM, et al Coronary Plaque Morphology and the Anti-Inflammatory Impact of Atorvastatin: A Multicenter 18F-Fluorodeoxyglucose Positron Emission Tomographic/Computed Tomographic Study. Circulation Cardiovascular Imaging. 2016;9(12):e004195 doi: 10.1161/CIRCIMAGING.115.004195 27956407 10.1161/CIRCIMAGING.115.004195 PMC5175997

[24] Joseph P, Ishai A, Mani V. Kallend D, Rudd JH, Fayad ZA, et al Short-term changes in arterial inflammation predict long-term changes in atherosclerosis progression. Eur J Nucl Med Mol Imaging. 2017;44(1):141–150. doi: 10.1007/s00259-016-3524-0 27738728 10.1007/s00259-016-3524-0

